# Supervised tele-mental health training in Ecuador during COVID-19: stress and coping among psychology students

**DOI:** 10.3389/fpsyg.2026.1905033

**Published:** 2026-08-18

**Authors:** Rafael Sánchez-Puertas, Byron Fernando Bustamante-Granda, Claudia Torres-Montesinos, María Belén Paladines-Costa, Marina R. Ramírez

**Affiliations:** 1 Departamento de Psicología, Universidad Técnica Particular de Loja, Loja, Ecuador; 2 Grupo de Investigación Bienestar, Salud y Sociedad, Escuela de Psicología y Educación, Universidad de Las Américas, Quito, Ecuador

**Keywords:** coping strategies, COVID-19, health professions education, perceived stress, psychology training, supervised practice, tele-mental health

## Abstract

**Objective:**

To evaluate perceived stress among senior psychology students delivering supervised tele-mental health services during the COVID-19 emergency in Ecuador, and to identify the coping strategies and contextual perceptions associated with variations in stress levels.

**Methods:**

A cross-sectional, observational study was conducted with 308 psychology trainees who completed a 30-h national training program and subsequently provided supervised telecare during the public health emergency. Perceived stress was measured using the 10-item Perceived Stress Scale (PSS-10), coping strategies via the Spanish Brief COPE, and sociodemographic attributes, perceived telecare self-efficacy, and perceived risk of COVID-19 contagion through *ad hoc* questionnaires. Data analysis comprised descriptive statistics, Spearman correlations, sex-based *t*-tests with Cohen's d, and robust hierarchical multiple linear regression with 5,000 bootstrap resamples and 95% bias-corrected and accelerated confidence intervals.

**Results:**

Mean perceived stress was 14.6/40, below the midpoint of the PSS-10. Stress was inversely associated with age (rho = −0.231) and perceived telecare self-efficacy (rho = −0.309) and positively associated with perceived COVID-19 contagion risk (rho = 0.195). Eleven coping strategies correlated significantly with stress, with the strongest associations observed for self-blame (rho = 0.587) and behavioral disengagement (rho = 0.409). Men reported greater use of humor, whereas women reported greater behavioral disengagement. The final regression model explained 52.2% of the variance in perceived stress and was positively associated with: self-blame, venting, self-distraction, denial, and perceived COVID-19 contagion risk, whereas religion and acceptance were negatively associated with stress.

**Conclusions:**

Supervised tele-mental health training in crisis contexts should include systematic monitoring of trainee stress, perceived vulnerability, and coping patterns. Educational supervision should strengthen adaptive coping, psychological flexibility, and timely emotional support for trainees involved in emergency telecare, particularly in resource-constrained health systems where students may be mobilized rapidly during severe public health emergencies and future disasters. These findings offer practical guidance for future emergency education training protocols.

## 1 Introduction

The COVID-19 pandemic forced health systems and universities to reorganize mental health care with exceptional speed. In Ecuador, as in other countries in the Global South, the national public health emergency and mandatory confinement led to the rapid virtualization of mental health services through telecare protocols (Ministerio de Salud Pública, 2020). At the same time, increased demand for psychological support and limited availability of qualified professionals required the emergency involvement of senior psychology students trained to provide supervised care in crisis settings (Ministerio de Salud Pública, 2020; Universidad Técnica Particular de Loja, 2021).

Consequently, the pandemic represented a turning point for the development of tele-mental health, imposing novel demands on universities, health systems, supervisors, and trainees. Perle et al. (2024) argue that involving psychology trainees, including doctoral trainees, requires deliberate reflection and evaluation by universities, particularly when training occurs in emergency contexts. Nevertheless, few studies have examined how these formative experiences affected trainees themselves, and even fewer have evaluated such experiences in countries of the Global South.

Psychology trainees recruited to respond to crises are a potentially vulnerable group in terms of mental health. To practice safely, they require adequate training and supervision that allow them to uphold professional ethical principles and codes of conduct (American Psychological Association, 2010), as well as context-specific regulations and protocols generated for each emergency. In Ecuador, universities, supervisors, and trainees had to learn, in a short period, a specific protocol and a set of telecare competencies that enabled them to comply with national guidelines (Ministerio de Salud Pública, 2020; Universidad Técnica Particular de Loja, 2021). These features of emergency telemental health create meaningful stressors for trainees and for the university system, raising challenges related to competency development, remote supervision, ethical care for service users, and emotional protection for trainees, supervisors, and users (Bergvall et al., 2023; Kule et al., 2026; Perle et al., 2024; Xing et al., 2023).

Although the analysis of trainees' coping strategies in critical situations has been recommended to improve planning for future emergencies, crises, and disasters (Schneider et al., 2020), the psychological impact of this type of supervised practice has rarely been explored. Evidence remains limited regarding the coping resources used by psychology students to face the simultaneous challenges imposed by the pandemic, telecare, and academic demands (Bergvall et al., 2023; Perle et al., 2024; Son et al., 2020).

Telecare itself is an important stressor to examine. Monaghesh and Hajizadeh (2020) define telehealth as the provision of health services using information and communication technologies for the safe and appropriate exchange of clinical information. During crises and disasters, universities incorporated telecare training into curricula, community engagement projects, and service-learning initiatives. However, these processes did not always evaluate their impact on students who participated in emergency services, nor did they systematically include trainees' perspectives to improve training or reduce the impact on their health and wellbeing (Jumreornvong et al., 2020; Schneider et al., 2020). The debate also remains open regarding the mental health risks of clinical practice environments and supervisory pedagogies in hospitals and health-related university faculties in Latin America (León-Rojas, 2026).

It is therefore necessary to improve both face-to-face and virtual training for telecare provision and to develop interventions that optimize stress-coping strategies among trainees (Amanvermez et al., 2020; Benjet, 2020; Dopp et al., 2021). Positive attitudes and self-confidence in performing clinical tasks have been described as protective factors for the mental health of health professionals (Babore et al., 2020; de la O-Martínez et al., 2024). Likewise, promoting positive attitudes and an internal locus of control among young people, so that they perceive themselves as capable of modifying their circumstances through their own efforts, can reduce psychological distress (Flesia et al., 2020). Training in tele-mental health still requires deeper reflection on how trainees' soft skills should be developed in this emerging specialist area (Dopp et al., 2021). For this reason, it is important to evaluate how psychology trainees coped with stressful situations and which factors influenced their mental health, particularly in experiences developed in low- and middle-income settings.

University students are a vulnerable group in terms of mental health during crises. Ecuadorian university students, like students in other countries, have been described as vulnerable to mental health problems (Torres et al., 2017), and stress has been associated with higher levels of anxiety, depression, alcohol use, and lower resilience (Ruisoto et al., 2020). International studies have shown that during social crises, including pandemics and armed conflicts, university students report increased perceived stress (Aslan et al., 2020; Bisht et al., 2021; Bourion-Bédès et al., 2021) and reduced psychological wellbeing (Son et al., 2020).

This vulnerability may be explained by changes in routines and study time (Aslan et al., 2020; Aucejo et al., 2020), fear of personal consequences or harm to loved ones (Son et al., 2020; Yang et al., 2021), difficulties adapting to political decisions such as social distancing, confinement, the mandatory transition to online classes, or technostress (Benjet, 2020; Bisht et al., 2021), increased uncertainty about the future (Benjet, 2020), and loneliness derived from reduced social bonds and diminished support from peers, teachers, and authorities (González-Sanguino et al., 2021; Kleiman et al., 2020).

Sociodemographic variables have also been associated with greater vulnerability to stress among university students facing crises and disasters. Several studies have reported that women, compared with men, experience poorer mental health consequences (Abuhmaidan and Al-Majali, 2020; Aslan et al., 2020; Bourion-Bédès et al., 2021) and significantly higher stress levels (Flesia et al., 2020; Lai et al., 2020; Wannapaschaiyong and Kallawicha, 2023). Sex-based differences have also been reported among Ecuadorian university students outside crisis periods (Ruisoto et al., 2020). In addition, students younger than 20 years have shown poorer mental health than older students (Abuhmaidan and Al-Majali, 2020).

Because social crises can increase stress among university students, it is necessary to examine how students cope with elevated stress. Coping strategies are efforts to regulate emotions, behaviors, cognitions, and environmental demands in response to daily and exceptional stressors (Morales-Rodríguez and Pérez-Marmol, 2019). Rice et al. (2014) distinguishes emotion-focused strategies, such as acceptance, emotional support, humor, positive reframing, and religion; problem-focused strategies, such as active coping, planning, and instrumental support; and dysfunctional strategies, such as denial, self-distraction, substance use, behavioral disengagement, self-blame, and venting. Yan et al. (2021) distinguishes positive strategies, including active coping, emotional support, instrumental support, and positive reframing, from negative strategies, including substance use, self-distraction, behavioral disengagement, and self-blame.

In Ecuadorian health professionals, active coping and social support have been significantly associated with lower psychological distress and better mental health outcomes (Ruisoto et al., 2021). This suggests that training programs should strengthen these strategies in future professionals. In contrast, inflexible and avoidant coping strategies may increase the likelihood of high stress and poorer mental health (Flesia et al., 2020; Ortega-Jiménez et al., 2021; Smith et al., 2020; Yu et al., 2020).

Studies with university students have reported that individuals with higher perceived stress during crises tend to use emotion-focused and problem-focused coping strategies (Rogowska et al., 2020). Increased alcohol use has also been documented as a coping strategy in response to stress (Browning et al., 2021; Charles et al., 2021), representing a risk factor in crisis contexts and underscoring the need for educational and preventive interventions (Charles et al., 2021; Jaffe et al., 2021). Maladaptive strategies such as alcohol use and avoidance have also been associated with the development and maintenance of psychological problems among Ecuadorian university students (López-Guerra et al., 2018; Torres et al., 2017). Identifying these coping patterns is therefore essential for designing training and health-promotion programs that strengthen resilience and wellbeing (Amanvermez et al., 2020; Benjet, 2020; Rogowska et al., 2020; Wannapaschaiyong and Kallawicha, 2023), including interventions that improve communication strategies among psychology trainees and reduce silence as an avoidant coping behavior during supervision (Marinho-Costa et al., 2023).

Despite this evidence, little is known about the coping strategies used by psychology students while delivering tele-mental health services in crisis contexts such as the COVID-19 pandemic in the Global South. The aim of this study was therefore to determine the level of perceived stress among psychology students who completed supervised telemental health training and to identify coping strategies associated with higher or lower stress.

## 2 Materials and methods

### 2.1 Study design and ethical considerations

A cross-sectional, observational study with a quantitative and correlational scope was conducted. Participation was confidential and anonymous. The study was approved by the Expedited Human Research Ethics Committee of the Ministry of Public Health of Ecuador (Code 009-2020) and was conducted in accordance with the principles of the Declaration of Helsinki. Written informed consent was obtained from all participants.

### 2.2 Participants

A non-probability convenience sampling strategy was used. Based on an a priori power calculation, a minimum sample size of 297 supervised trainees who had provided tele-mental health support during the COVID-19 public health emergency in Ecuador was required to achieve a 95% confidence level with a 3% margin of error. A total of 621 senior psychology students from across the country were invited to participate, and 373 accepted the invitation, corresponding to an acceptance rate of 60.06%. Inclusion criteria were being a final-year psychology student, being of legal age, having completed and passed the 30-h training program in telecare, and providing informed consent. After data cleaning, the final analytic sample included 308 supervised trainees. The mean age was 26.68 years (SD = 6.71; range 20–54). Three quarters of the participants were women, single, and from the provinces of Guayas, Manabí, Pichincha, and Azuay, most attended public universities. This distribution reflects the typical profile of final-year psychology students in Ecuador (see Table 1).

**Table 1:** Sociodemographic characteristics of participants.

Variable	*M*	Range	SD
Age	26.68	20–54	6.71
**Variable**	**n**	**%**	
Sex: male	74	24.0	
Sex: female	234	76.0	
Province: Guayas	95	31.1	
Province: Manabí	58	19.0	
Province: Pichincha	48	15.7	
Province: Azuay	23	7.5	
Province: rest of country	81	26.7	
Marital status: single	229	74.4	
Marital status: married/common-law union	68	22.1	
Marital status: divorced/separated	11	3.5	
University: public	252	83.2	
University: private	51	16.8	

Missing values for province = 3; Missing values for type of university = 5.

SD, Standard Deviation.

### 2.3 Instruments

The *ad hoc* sociodemographic questionnaire collected information on sex, age, province, marital status, ethnicity, socioeconomic level, and type of university. A second *ad hoc* questionnaire assessed training- and intervention-related factors, including perceived self-efficacy for telecare and perceived risk of becoming a victim of the COVID-19 crisis. These *ad hoc* surveys were designed by the research team and validated by the MSP technical team that participated in the design of the research protocol. It was decided to measure perceptions through two questions in which practitioners self-report the perception of self-efficacy and risk of COVID-19 using a scale of 1 to 10 levels to increase the possibility of variability in responses. In addition, the option of evaluating through two questions and not through specific questionnaires was chosen, to avoid creating a long protocol that reduces the number of subjects evaluated or increases the lost data. Content validity of the two *ad hoc* items was established through expert review by the Ministry of Public Health technical team. A pilot test with 10 psychology students confirmed item clarity and comprehension before full-scale implementation.

Coping strategies were assessed using the Spanish version of the Brief COPE by Morán et al. (2010). This 28-item instrument evaluates 14 coping strategies: acceptance, emotional support, humor, positive reframing, religion, active coping, planning, instrumental support, denial, self-distraction, substance use, behavioral disengagement, self-blame, and venting. Participants responded on a four-point scale ranging from 0, I have not been doing this at all, to 3, I have been doing this a lot. Subscale scores range from 0 to 6, with higher scores indicating greater use of each coping strategy. The internal consistency of its factors, including cognitive (i.e., positive reframing, active coping, planning, behavioral disengagement), social (i.e., emotional support, instrumental support, and venting), avoidant (i.e., humor, denial, self-distraction, substance use, and self-blame), and religious strategies, has been reported with Cronbach's alpha values ranging from 0.74 to 0.80. In this cohort the Cronbach's alpha value was 0.63 for social strategies, 0.69 for cognitive strategies and 0.76 for avoidant strategies, in religion and acceptance the Cronbach's alpha values were < 0.3. However, the second-order factor structure reported by Morán et al. (2010) is not universally accepted, so it is preferable to use the 14-factor first-order factor structure proposed by Carver (1997).

Perceived stress was measured using the 10-item Perceived Stress Scale (PSS-10), in the Spanish version validated for Ecuador by Ruisoto et al. (2020). The scale assesses the extent to which individuals perceive lack of control in daily life. Responses are recorded on a five-point Likert scale from 0, never, to 4, very often. Total scores range from 0 to 40, with higher scores indicating greater psychological stress. Internal consistency in the Ecuadorian population was Cronbach's alpha = 0.80. In this cohort the Cronbach's alpha value was = 0.89.

### 2.4 Procedure

The supervised trainees completed a 30-h training self-instructional online program (Universidad Técnica Particular de Loja, 2021) between April 1 and May 31, 2020, the objective of the training was to provide the student with the skills required to join the mental health telecare work team of the MSP; seven training modules were given: (**I):** Knowledge of the mental health telecare protocol of the MSP, **(II):** Telecare, (**III):** Crisis intervention, psychological first aid, (**IV):** Grief management, **(V):** Management of intervention and self-care teams, **(VI):** Accompaniment in mental disorders—management and reduction of risks in substance use, **(VII):** Information management in crisis. By passing the course, the student would be able to perform psychoeducation, emotional containment and psychological first aid, according to the MSP's mental health telecare protocol. Once the course was approved, they were gradually incorporated into the telecare protocol between April 13 and May 31.

The telemental health intervention program began on March 24, 2020, initially involving only licensed psychologists and psychiatrists, and continued throughout the COVID-19 lockdown period. The present study focuses on the first cohort of psychology students who joined the program during the most critical phase of the pandemic in Ecuador (April to September 2020). Before joining the program, none of the students had previous experience in tele-psychological care, psychological crisis intervention, or emergency response. Each student provided up to 2 h of telemental health assistance per session, three times per week. Their responsibilities included psychoeducation, emotional support, psychological first aid, and referral of cases requiring specialized care to psychologists and psychiatrists participating in the Ministry of Public Health (MSP) protocol (Ministerio de Salud Pública, 2020).

Clinical supervision was provided by faculty members appointed by each participating university and delivered entirely through tele-supervision. Supervisors remained connected in real time during telecare sessions, enabling continuous monitoring, immediate feedback, and timely support for trainees when complex clinical situations arose. Furthermore, structured group supervision sessions were conducted weekly to facilitate case discussions, review clinical decision-making processes, address ethical and operational issues, and encourage reflective practice. This supervisory framework sought to ensure adherence to the Ministry of Public Health protocol, maintain service quality, safeguard trainee and patient welfare, and strengthen professional competencies in emergency tele-mental health care.

Data were collected between September 11, 2020, and January 15, 2021, once they completed their academic cycle of supervised internships. The Ministry of Public Health of Ecuador emailed the research invitation to all students who had provided telecare within the yellow-code pathway of the national emergency mental health protocol during the COVID-19 pandemic (Ministry of Public Health, 2020). Trainees freely accepted the informed consent form and then accessed an online psychological battery composed of two *ad hoc* questionnaires and two psychological tests, requiring approximately 30 min to complete. No question was mandatory, and no financial or academic compensation was offered. At the end of participation, each trainee received a summary of their scores and could request a feedback session if desired. In addition, since the data collection was anonymous, it was not possible to identify students with high stress scores during data collection, but our team provided them with psychoeducational material to reduce stress, along with the report, and they were also motivated to access the student welfare services of their respective universities and to use the 171 option 6 line that the MSP enabled to receive free care.

### 2.5 Data analysis

After data cleaning, descriptive statistics were calculated using measures of central tendency and dispersion for continuous variables, and frequencies and percentages for categorical variables. Descriptive analyses were conducted separately for trainees with higher stress (PSS-10 > 15) and lower stress (PSS-10 < 15).

Before conducting bivariate analyses, normality of continuous variables was assessed with the Shapiro-Wilk test. Age, the 14 coping strategies, perceived self-efficacy for telecare, and perceived risk of COVID-19 contagion showed significant deviations from normality (*p* < 0.05). Accordingly, non-parametric tests were used for these variables. Perceived stress, the dependent variable, showed a normal distribution (Shapiro-Wilk *p* = 0.067), unlike the independent variables described above.

Spearman's correlation coefficient (rho) was used to examine associations between perceived stress and age, perceived self-efficacy for telecare, perceived risk of COVID-19 contagion, and the 14 coping strategies. Sex-based differences were analyzed with Student's *t* test, and effect sizes were estimated using Cohen's d. Statistical significance was set at *p* < 0.05 for all inferential analyses.

To examine multivariable associations, robust hierarchical multiple linear regression models were estimated using 5,000 bootstrap resamples and 95% bias-corrected and accelerated confidence intervals. This approach was selected because several variables deviated from normality, and bootstrap estimation provides more stable parameter estimates under these conditions. The dependent variable was perceived stress. The independent variables were sex, age, perceived self-efficacy for telecare, perceived risk of COVID-19 contagion, and the 14 coping strategies. It was decided to include the 14 strategies simultaneously, because the Brief COPE was originally designed as 14 distinct coping dimensions, and not universally accepted higher-order factorial structure has been established, leading researchers to evaluate alternative grouping models across samples (Carver, 1997). Previous research has shown substantial inconsistency in the factorial structure of the Brief COPE across populations and cultural contexts. A systematic review identified factorial solutions ranging from 2 to 15 factors and found little consensus regarding a universal higher-order structure (Solberg et al., 2022). More recent evidence suggests that the original 14 correlated coping dimensions proposed by Carver (1997) may offer the most psychometrically robust representation of the instrument, although higher-order models remain under debate (Rodrigues et al., 2022).

The independent variables selection followed a systematic and theory-informed approach aimed at model parsimony. In the first layer, the two demographic variables were entered. In the second layer, the two perception variables were added. In the third layer, the 14 coping strategies were introduced. Prior to modeling, regression assumptions were evaluated through residual histograms and probability-probability plots for residual normality, standardized residual plots for homoscedasticity, and the Durbin-Watson statistic for independence of errors. Durbin-Watson values between 1.5 and 2.5 were considered acceptable. Multicollinearity was assessed by inspecting the correlation matrix among predictor variables and by calculating variance inflation factor (VIF) values. No excessively high correlations were observed among the predictors, all tolerance values were superior 0.2 and all VIF values were below 5, indicating that multicollinearity was not a concern. Model fit was assessed using R^2^, adjusted R^2^, and the global F test. The contribution of each independent variable was examined using standardized coefficients, *t* values, and *p* values. The final model was refined to include statistically significant factors only. Analyses were conducted in IBM SPSS Statistics 25 and Jamovi 2.7.

## 3 Results

Mean perceived stress was 14.6 out of 40, below the midpoint of the PSS-10 scale. Regarding factors associated with the COVID-19 health crisis during the telecare process, perceived self-efficacy for telecare had a mean score of 7.74 out of 10, above the midpoint of the scale, indicating a generally positive sense of competence. Perceived risk of COVID-19 contagion was slightly above the midpoint, with a mean score of 5.56 out of 10 (see Table 2).

**Table 2:** Factors associated with perceived stress.

Variable	*M*	Range	SD
Perceived stress (*n* = 308)	14.6	0–40	6.98
Perceived self-efficacy for telecare (*n* = 308)	7.74	1–10	1.83
Perceived risk of COVID-19 contagion (*n* = 308)	5.56	1–10	2.29

SD, Standard Deviation.

The most frequently used coping strategies among supervised trainees were two emotion-focused strategies, acceptance and positive reframing, followed by two problem-focused strategies, active coping and planning. The main dysfunctional strategies were self-distraction and self-blame (see Table 3).

**Table 3:** Coping strategies reported by supervised trainees.

Coping strategy	*M*	Missing values	Range	SD
Self-distraction	2.74	3	0–6	1.62
Active coping	2.99	2	0–6	1.59
Denial	0.49	2	0–6	0.98
Substance use	0.28	3	0–6	0.86
Emotional support	1.78	2	0–6	1.71
Instrumental support	1.80	2	0–6	1.50
Behavioral disengagement	0.97	2	0–6	1.19
Venting	1.78	2	0–6	1.44
Positive reframing	2.79	2	0–6	1.67
Planning	2.81	6	0–6	1.49
Humor	0.97	2	0–6	1.26
Acceptance	3.87	2	0–6	1.60
Religion	2.16	2	0–6	1.50
Self-blame	1.25	2	0–6	1.49

SD, Standard Deviation.

Perceived stress was moderately and inversely correlated with age (rho = −0.231) and perceived self-efficacy for telecare (rho = −0.309). It was weakly and positively correlated with perceived risk of COVID-19 contagion (rho = 0.195). In other words, trainees who perceived greater stress during tele-mental health practice tended to be younger, to feel less capable of providing telecare, and to perceive greater risk of becoming victims of COVID-19 (see Table 4).

**Table 4:** Factors associated with stress among supervised trainees.

Variable	Low stress (PSS-10<15) (*n*=162)		High stress (PSS-10 > 15) (*n* = 146)			
	M (SD)	Missing values	M (SD); *n*	Missing values	Spearman's rho	*p*
Age	27.98 (7.81)	0	25.25 (4.86)	0	−0.231	< 0.001
Perceived telecare self-efficacy	8.08 (1.72)	0	7.38 (1.89)	0	−0.309	< 0.001
Perceived COVID-19 contagion risk	5.11 (2.15)	0	6.06 (2.36)	0	0.195	< 0.001
Self-distraction	2.46 (1.64)	2	3.05 (1.53)	1	0.239	< 0.001
Active coping	2.96 (1.63)	0	3.02 (1.54)	2	0.016	0.780
Denial	0.25 (0.59)	0	0.76 (1.22)	2	0.296	< 0.001
Substance use	0.13 (0.50)	2	0.44 (1.11)	1	0.238	< 0.001
Emotional support	1.34 (1.44)	1	2.31 (1.84)	1	0.388	< 0.001
Instrumental support	1.61 (1.42)	1	1.01 (1.55)	1	−0.219	< 0.001
Behavioral disengagement	0.61 (0.96)	1	1.37 (1.29)	1	0.409	< 0.001
Venting	1.46 (1.32)	1	2.13 (1.48)	1	0.356	< 0.001
Positive reframing	2.82 (1.71)	1	2.76 (1.63)	1	−0.019	0.738
Planning	2.62 (1.48)	2	3.04 (1.48)	4	0.159	0.006
Humor	0.86 (1.23)	1	1.11 (1.29)	1	0.165	0.004
Acceptance	3.96 (1.60)	1	3.78 (1.60)	1	−0.091	0.113
Religion	2.01 (1.28)	1	2.32 (1.70)	1	0.160	0.005
Self-blame	0.59 (0.88)	1	1.98 (1.68)	1	0.587	< 0.001

Values for the low- and high-stress groups are presented as M (SD); n values are reported within each cell.

p values are based on Spearman correlations.

SD, Standard Deviation.

When correlations between perceived stress and coping strategies were examined, 11 of the 14 strategies were significantly associated with stress. Moderate positive correlations were observed for self-blame (rho = 0.587) and behavioral disengagement (rho = 0.409). Weak positive correlations were observed for emotional support (rho = 0.388), venting (rho = 0.356), denial (rho = 0.296), self-distraction (rho = 0.239) and substance use (rho = 0.238). Very weak positive correlations were observed for humor (rho = 0.165), religion (rho = 0.160), and planning (rho = 0.159). And weak positive correlation was observed for instrumental support (rho = −0.219) (see Table 4).

Sex-based analyses showed no significant difference in perceived stress between women and men (women: *M* = 14.98, SD = 6.83; men: *M* = 13.44, SD = 7.39; *t*[306] = 1.66, *p* = 0.099). However, two coping strategies differed by sex. Men reported greater use of humor (women: *M* = 0.81, SD = 1.14; men: *M* = 1.48, SD = 1.49; *t*[306] = −4.01, *p* < 0.001), with a medium effect size (Cohen's d = 0.537). Women reported greater use of behavioral disengagement (women: *M* = 1.05, SD = 1.19; men: *M* = 0.73, SD = 1.16; *t*[306] = 2.02, *p* = 0.044), with a small effect size (Cohen's *d* = 0.271).

The robust hierarchical regression analyses evaluated the predictive value of demographic variables, perceived self-efficacy for telecare, perceived risk of COVID-19 contagion, and coping strategies on perceived stress. In the first layer, which included sex and age, the model was statistically significant, *F*(2, 298) = 12.45, *p* < 0.001, and explained 7.8% of the variance in perceived stress (*R*^2^ = 0.078; adjusted *R*^2^ = 0.071). Age was a significant negative association (beta = −0.265, *p* < 0.001) (See Table 5).

**Table 5:** Robust hierarchical multiple regression predicting perceived stress scores among psychology trainees providing telecare during the COVID-19 pandemic.

Layer	Factor	*B*	Bootstrap SE	Beta	*t*	*p*	95% CI	Tolerance	VIF
Layer 1	Age	−0.283	0.060	−0.265	−4.738	< 0.001	−0.401/−166	0.995	1.005
Layer 2	Age	−0.239	0.058	−0.224	−4.132	< 0.001	−0.353/−0.125	0.965	1.036
	Perceived self-efficacy for telecare	−0.835	0.211	−0.218	−3.955	< 0.001	−1.25/−0.419	0.932	1.073
	Perceived COVID-19 contagion risk	0.540	0.166	0.176	3.256	0.001	0.214/0.866	0.977	1.024
Layer 3	Self-blame	2.109	0.261	0.449	8.074	< 0.001	1.595/2.623	0.551	1.815
	Venting	0.843	0.275	0.173	3.066	0.002	0.302/1.384	0.534	1.837
	Self-distraction	0.618	0.230	0.141	2.690	0.008	0.166/1.071	0.619	1.615
	Denial	0.876	0.354	0.122	2.472	0.014	0.178/1.574	0.702	1.424
	Perceived COVID-19 contagion risk	0.357	0.131	0.116	2.726	0.007	0.099/0.615	0.940	1.064
	Religion	−0.655	0.309	−0.139	−2.120	0.035	−1.264/−0.047	0.398	2.510
	Acceptance	−0.549	0.243	−0.125	−2.255	0.025	−1.028/−0.070	0.560	1.787
	Constant	14.271	2.297		6.214	< 0.001	9.759/18.792		

B = unstandardized regression coefficient; beta = standardized regression coefficient; SE = standard error; CI = confidence interval; VIF = variance inflation factor.

Bootstrap estimates were calculated using 5,000 resamples with 95% bias-corrected and accelerated confidence intervals.

In the second layer, perceived self-efficacy for telecare and perceived COVID-19 risk were added. The model remained statistically significant, *F*(4, 298) = 14.52, *p* < 0.001, and explained 16.5% of the variance (*R*^2^ = 0.165; adjusted *R*^2^ = 0.154). Age remained a significant negative association (beta = −0.224, *p* < 0.001), perceived self-efficacy for telecare was also a significant negative association (beta = −0.218, *p* < 0.001), and perceived COVID-19 risk was a significant positive association (beta = 0.176, *p* = 0.001) (See Table 5).

In the third layer, the 14 coping strategies were added. The model was statistically significant, *F*(18, 298) = 16.99, *p* < 0.001, and explained 52.2% of the variance in perceived stress (*R*^2^ = 0.522; adjusted *R*^2^ = 0.491). The Durbin-Watson statistic was 2.090, indicating no problematic autocorrelation of residuals. Positive association of perceived stress were self-blame (beta = 0.449, *p* < 0.001), venting (beta = 0.173, *p* = 0.002), self-distraction (beta = 0.141, *p* = 0.008), denial (beta = 0.122, *p* = 0.014), and perceived risk of COVID-19 contagion (beta = 0.116, *p* = 0.007). Negative association were religion (beta = −0.139, *p* = 0.035) and acceptance (beta = −0.125, *p* = 0.025) (See Table 5).

## 4 Discussion

This study examined perceived stress among psychology students who completed supervised tele-mental health training and provided telecare during the COVID-19 public health emergency in Ecuador. It also identified coping strategies associated with perceived stress. The findings offer evidence relevant to medical and health professions education in crisis contexts, particularly for universities in resource-constrained settings where trainees may be rapidly mobilized to support overstretched health systems.

The mean perceived stress score was 14.6, below the midpoint of the PSS-10. This indicates relatively low perceived stress, especially when compared with previously reported values among Ecuadorian university students, whose mean approached the midpoint of the PSS-14 (Ruisoto et al., 2020). It was also lower than values reported among university students in the United Kingdom, the United States, France, Poland, and Turkey during similar crisis contexts assessed with the PSS-10 (Aslan et al., 2020; Bourion-Bédès et al., 2021; Lai et al., 2020; Rogowska et al., 2020). These findings indicate that senior psychology trainees may have experienced lower stress than expected at the end of their internship, despite the expectation that emergency tele-mental health practice would increase perceived stress. According to Lazarus and Folkman's (1984) transactional model, stressful experiences are mediated by cognitive appraisal processes rather than determined solely by objective circumstances. Although the COVID-19 pandemic constituted a major environmental stressor, trainees may have come to perceive telehealth practice as a challenge rather than a threat as they gained experience and received supervisory support. This change in appraisal, together with the development of coping resources, may help explain why perceived stress levels were lower than expected despite the adverse circumstances.

One factor expected to be associate with stress level was the high perceived self-efficacy for telecare reported by participants. The mean score of 7.74 out of 10 may be related to the 30-h training program required for inclusion in the study (Universidad Técnica Particular de Loja, 2021) and to the supervision and support process implemented in the national protocol (Ministerio de Salud Pública, 2020). From the perspective of Lazarus and Folkman's (1984) transactional model, self-efficacy can be understood as a personal coping resource that influences secondary appraisal processes, that is, individuals' evaluation of their ability to manage situational demands. Higher perceived self-efficacy may therefore have reduced the likelihood of appraising tele-mental health activities as overwhelming or threatening. Consistent with this interpretation, perceived self-efficacy for telecare showed a significant association with perceived stress in the bivariate analyses and in the first two regression models. However, after coping strategies were included, its contribution was no longer significant, suggesting that the influence of self-efficacy may operate indirectly through the selection and implementation of coping responses. In other words, students who perceived themselves as more capable may have been more likely to engage in adaptive coping strategies, which in turn were more directly associated with lower levels of perceived stress. This partially challenges previous evidence on the protective role of positive attitudes and self-efficacy (Babore et al., 2020; de la O-Martínez et al., 2024; Flesia et al., 2020) and suggests that coping skills may represent a more proximal and pedagogically modifiable target for universities.

From an educational planning perspective, these findings support the need to strengthen tele-mental health training in emergencies while considering age, sex, and perceived crisis-related risk. In bivariate analyses and in the first two regression layers, younger trainees reported higher perceived stress, although age was no longer significant after coping strategies were included. This pattern is consistent with previous research describing poorer mental health among younger university students (Abuhmaidan and Al-Majali, 2020; Torres et al., 2017). Universities should therefore provide specific spaces for strengthening soft skills among less experienced trainees and should use qualitative research to explore why younger students may be more vulnerable during emergency practice.

Contrary to several studies conducted with university students during COVID-19 (Flesia et al., 2020; Lai et al., 2020; Wannapaschaiyong and Kallawicha, 2023) and with Ecuadorian students outside crisis periods (Ruisoto et al., 2020), perceived stress did not differ significantly by sex. In the regression models, the inclusion of cognitive perceptions, age, and coping strategies suppressed any baseline sex-related differences in explaining perceived stress. It is possible that training and supervisory support reduced expected sex differences, or that the pandemic generated conditions that were broadly similar across the university population.

Nevertheless, sex differences in coping strategies were identified. Women reported greater behavioral disengagement, a strategy generally considered dysfunctional. Future supervision processes should include actions to reduce behavioral disengagement, particularly among female trainees. This implies discouraging avoidance of stressors, since avoidant strategies are inefficient and have been associated with poorer mental health (Flesia et al., 2020; López-Guerra et al., 2018; Ortega-Jiménez et al., 2021; Smith et al., 2020; Torres et al., 2017; Yu et al., 2020). One possible explanation for the higher levels of behavioral disengagement observed among women relates to contextual and sociocultural factors that were not assessed in this study. During the COVID-19 pandemic, female students may have faced a greater burden of caregiving, household, and academic responsibilities, consistent with traditional gender-role expectations prevalent in many Latin American contexts and these additional demands may have increased vulnerability to stress (Ferigra-Stefanović, 2023). Alternatively, behavioral disengagement may also reflect a temporary withdrawal from action as a means of seeking rest, reducing emotional overload, or preserving psychological resources when faced with multiple simultaneous demands. Future research should explore the influence of gender roles, family responsibilities, caregiving demands, and the potential restorative function of behavioral disengagement on coping strategies among psychology trainees in crisis contexts.

These findings suggest that universities should incorporate a gender-sensitive perspective into the design of supervised practice, especially in programs with high female participation, such as psychology. This is relevant to telecare because telehealth has been proposed as a mechanism for reducing emotional exhaustion among women and for improving labor equity in the health professions (Nadkarni and Mittal, 2021). Future research should explore the mechanisms explaining the greater use of behavioral disengagement among women, with the aim of improving trainees' ability to manage stress.

On the other hand, men reported greater use of humor, which may function as a protective coping strategy. Humor has also been described as beneficial for health professionals involved in care provision (Losada and Lacasta, 2019). From a sociocultural perspective, this finding may reflect gender norms commonly described in Latin American contexts, where men are often encouraged to maintain emotional control and limit overt expressions of vulnerability (Kotthoff, 2022; Ojeda and Liang, 2014). Within this framework, humor may provide a socially acceptable means of expressing discomfort, managing stress, and fostering social cohesion while preserving masculine norms of emotional restraint.

Perceived risk of COVID-19 contagion was significantly associated with higher stress among trainees. This result is consistent with Yang et al. (2021) and with evidence of panic-related symptoms among Ecuadorian university students even outside crisis contexts (Torres et al., 2017). From the perspective of Lazarus and Folkman's (1984) transactional model, perceived risk can be understood as part of the primary appraisal process, through which individuals evaluate the potential threat posed by an event. Trainees who perceived a greater likelihood of infection may have appraised the clinical and telehealth context as more threatening, thereby increasing their stress responses. This finding suggests that stress was influenced not only by the objective presence of the pandemic, but also by the subjective interpretation of its risks. Consequently, training programs should include emotional self-regulation skills and opportunities to reframe perceived threats, while establishing clear and timely mechanisms for emotional containment and immediate support within supervision protocols. Such mechanisms should be incorporated into the institutional commitments of both face-to-face and virtual practice settings, in accordance with professional and national regulations (American Psychological Association, 2010; Ministerio de Salud Pública, 2020).

Two coping strategies were significantly associated with lower perceived stress: religion and acceptance. Both can be understood as strategies that cognitively reframe the stressor (Carver, 1997). From the transactional model of stress and coping (Lazarus and Folkman, 1984), these strategies may reduce the perceived threat of a stressor by placing the event in a broader framework of meaning or by accepting the situation as it unfolds. The protective role of acceptance is consistent with research highlighting psychological flexibility as a key factor for university students' mental health (Flesia et al., 2020; Ortega-Jiménez et al., 2021; Smith et al., 2020; Yu et al., 2020), aligned with transdiagnostic approaches and third-wave behavioral therapies (López-Guerra et al., 2018). Future studies should qualitatively examine how these strategies can be developed through simulation, supervised co-therapy, and reflective practice. Although religion and acceptance emerged as significant variables in the final regression model, the reliability of these subscales was extremely low in the present sample (Cronbach's α < 0.30). Therefore, any interpretation of their protective role should be regarded as tentative. Given the substantial measurement limitations associated with such low reliability coefficients, these findings should not be considered definitive and warrant confirmation in future studies using instruments with stronger psychometric properties.

Four avoidant strategies were significantly associated with higher perceived stress: self-blame, venting, self-distraction, and denial. This is consistent with evidence suggesting that active coping and social support should be strengthened because both can mediate stress and burnout (Ruisoto et al., 2021; Yu et al., 2020). It is also consistent with studies showing the need to prevent avoidant and rigid coping styles among university students (Flesia et al., 2020; López-Guerra et al., 2018; Marinho-Costa et al., 2023; Ortega-Jiménez et al., 2021; Smith et al., 2020; Yu et al., 2020). From the perspective of Lazarus and Folkman's (1984) transactional model, these strategies may reflect attempts to regulate the emotional impact of a stressor without directly addressing the demands or modifying the appraisal of the situation. As a result, the discrepancy between perceived demands and available coping resources may persist, contributing to higher levels of perceived stress. Denial and self-distraction may hinder realistic appraisal and problem-solving efforts, whereas self-blame may intensify negative emotional reactions and perceptions of inadequacy. These findings suggest that avoidant coping strategies may be maladaptive in prolonged and uncertain contexts such as the COVID-19 pandemic, where sustained adaptation and cognitive reappraisal are required. For health professions education, this finding is highly actionable: supervisors can be trained to detect avoidant coping patterns and redirect students toward more adaptive, reflective, problem-focused and socially supported strategies.

Future studies should use pre-post intervention designs and longitudinal approaches to evaluate the long-term impact of coping strategies among mental health trainees working in crises, emergencies, and disasters. As noted by Amanvermez et al. (2020), Benjet (2020), and Rogowska et al. (2020), training in adaptive coping can strengthen health promotion, resilience, and wellbeing among both intervention providers and service users. This has important educational and social implications, because stress-reduction strategies among supervised trainees may protect them from worsening mental health and distress (León-Rojas, 2026). Universities should therefore continuously monitor stress and coping strategies throughout supervised practice, especially when students intervene in crisis contexts either online or in person.

This study has several limitations:

First, the use of convenience sampling limits the generalizability of the findings, as approximately 40% of eligible participants did not take part in the study. In addition, self-selection bias may have occurred, since students who were more motivated to participate may also have been those experiencing higher levels of stress or greater interest in learning about their results.

Second, the use of self-report measures may have introduced recall bias, subjective interpretation, and social desirability effects, despite the confidentiality and anonymity guaranteed to participants. Furthermore, data were collected approximately 6 months after the onset of the COVID-19 pandemic in Ecuador and upon completion of the clinical internship, meaning that responses may have reflected participants' overall retrospective experience rather than their immediate reactions during supervised telehealth activities. This timing may also have influenced perceived stress levels, as students may have partially adapted to pandemic-related restrictions and the telehealth modality, potentially resulting in different perceptions than those experienced during the initial stages of the health emergency.

Third, the cross-sectional design, together with the absence of pre- and post-placement assessments, precludes causal inferences and limits the ability to examine changes in perceived stress and coping strategies over time. In addition, stress levels may have been influenced by local pandemic conditions in the regions where trainees lived and worked. Although the study included participants from across Ecuador, the impact of COVID-19 varied substantially between regions. At the time of assessment, Guayas Province had experienced the most severe effects of the pandemic, while other regions faced different levels of transmission and healthcare burden. These contextual differences occurred within a national scenario characterized by increasing infections and mortality rates and a healthcare system under considerable strain, factors that may have influenced students' stress perceptions.

Fourth, potentially relevant covariates, such as prior mental health status, academic and clinical workload, quality of supervision, resilience, and social support, were not assessed and may have influenced both perceived stress and the coping strategies adopted by participants.

Finally, women represented a large proportion of the sample (76%), which may have influenced the findings related to coping strategies and gender comparisons. However, this distribution reflects the Ecuadorian context, where psychology programs are predominantly composed of female students. Therefore, findings regarding gender differences should be interpreted with caution and replicated in future studies using more balanced and randomly selected samples.

Despite these limitations, the study provides valuable evidence for improving the supervision and training of future psychology professionals, particularly in crisis situations and in developing-country settings.

In conclusion, this cohort of supervised psychology trainees reported lower levels of perceived stress than would typically be expected during a large-scale crisis such as the COVID-19 pandemic. Perceived stress was inversely related to age and perceived self-efficacy for telecare. The robust linear regression model explained 52.2% of perceived stress. Higher stress was associated with self-blame, venting, self-distraction, denial, and perceived risk of COVID-19 contagion; lower stress was associated with religion and acceptance.

The educational implications of these findings can be summarized in five recommendations for universities. First, training processes should increase trainees' awareness of their vulnerability when acting in crisis and emergency situations. Second, universities should assess trainee stress before, during, and after participation in mental health support services. Third, coping strategies should be evaluated during trainee selection and supervision, and the impact of supervised practice on these soft skills should be identified. Fourth, supervisors should be trained in early detection of avoidant coping strategies. Fifth, training programs should assess perceived risk and specific fears related to the crisis context trainees will face, enabling timely support that reduces the impact on their mental health during practice.

At the policy level, three recommendations emerge from these findings. First, health authorities and higher education regulators should establish minimum standards for the protection of trainees involved in telehealth and mental health services during crisis situations, including mandatory psychological risk monitoring and access to support resources. Second, accreditation and quality assurance frameworks should require structured supervision protocols that systematically assess stress, perceived risk, and coping processes throughout clinical training. Third, national regulatory and professional bodies should promote competency frameworks that incorporate self-care, resilience, coping skills, and psychological first aid as core components of professional preparation for crisis-response settings. Such measures may contribute to safeguarding trainee wellbeing while improving the quality and sustainability of mental health services delivered during emergencies.

Overall, the findings reaffirm the responsibility of universities and the health public system to anticipate the needs of vulnerable student populations and to improve the academic management of supervised practice. Future professionals should be able to provide mental health support during emergencies while also developing robust self-care skills.

## Data Availability

The datasets presented in this study can be found in online repositories. The names of the repository/repositories and accession number(s) can be found below: https://www.researchgate.net/publication/405521657_Base_consolidada_de_Practicantes_de_Psicologia_y_Psicologos_durante_del_Covid-19_en_Ecuador.

## In memoriam

In Memoriam of Dr. Rafael Sánchez-Puertas († April 29, 2026). The authors gratefully acknowledge his scientific contributions, dedication, and commitment to this research. His legacy will continue to inspire our academic work.
